# A comparison of methodologies for the staining and quantification of intracellular components of arbuscular mycorrhizal fungi in the root cortex of two varieties of winter wheat

**DOI:** 10.1099/acmi.0.000083

**Published:** 2019-11-21

**Authors:** Thomas I. Wilkes, Douglas J. Warner, Veronica Edmonds-Brown, Keith G. Davies, Ian Denholm

**Affiliations:** ^1^​ Department of Biological and Environmental Sciences, University of Hertfordshire, Hatfield, UK

**Keywords:** AM fungi, trypan blue, Sheaffer blue, wheat, staining

## Abstract

Arbuscular mycorrhizal (AM) fungi are one of the most common fungal organisms to exist in symbiosis with terrestrial plants, facilitating the growth and maintenance of arable crops. Wheat has been studied extensively for AM fungal symbiosis using the carcinogen trypan blue as the identifying stain for fungal components, namely arbuscles, vesicles and hyphal structures. The present study uses Sheaffer blue ink with a lower risk as an alternative to this carcinogenic stain. Justification for this is determined by stained wheat root sections (*n*=120), with statistically significant increases in the observed abundance of intracellular root cortical fungal structures stained with Sheaffer blue ink compared to trypan blue for both Zulu (*P*=0.003) and Siskin (*P*=0.0003) varieties of winter wheat. This new alternative combines an improved quantification of intracellular fungal components with a lower hazard risk at a lower cost.

## Introduction

Arbuscular mycorrhizal (AM) fungi are the most common fungal organisms to exist symbiotically with the root structures of vascular plants [1]. It is currently thought that the closely established relationship between plant and fungi contributed to early plant colonization of land [2]. To plants, AM fungi provide increases in nutrients and water through large branching mycelial networks and large surface areas resultant of intracellular components within root cortical cells [3]. In exchange, the plant provides photosynthetic products [4], such as carbohydrates [5]. Within arable farming, this aids the improved use of applied fertilizers, reduction of disease, resistance to salt stress and salinity, improved drought tolerance and improvements to crop quality [6].

Current staining procedures target arbuscules, vesicles and hyphal structures within the root cortex. Staining of target structures is performed for rapid, simple and cost-effective assessment of fungal symbiosis. Using light microscopy, the required skill sets are lower and the procedure can be performed with ease. Lactophenol cotton blue (C_37_H_27_N_3_Na_2_O_9_S_3_) is one of several stains that has been widely utilized for many years [7]. However, a move to the use of trypan blue (C_34_H_28_N_6_O_14_S_4_), originally developed by Philips and Hayman (1970) [8], has improved the clarity of characteristic AM fungal components [9]. To increase the selectiveness of trypan blue towards fungal root structures, the employment of a formaldehyde fixative solution to preserve plant tissue is required. A comparison of 14 different AM fungal staining methods developed between 1970 and 2014 typically used formaldehyde as a fixative solution to stabilize the plant cells in combination with trypan blue [10]. With the employment of a heat treatment, root fungal structures can be deliberately damaged to allow trypan blue to enter fungal cells. The fixing of plant tissues reduces damage from heat treatment, allowing trypan blue to have increased sensitivity towards intracellular fungal root components. Without formaldehyde, trypan blue would stain all cells in the sample, preventing the differentiation of individual fungal components. By selectively damaging the fungal structures, trypan blue becomes more effective.

Many widely used fungal root stains, including trypan blue, are known carcinogens [11]. As a consequence, many practitioners experienced in the biological staining of fungal root components have been searching for alternative dyes and stains. Trypan blue, however, is still a widely used stain for root cortical fungal structures. Tsaousis *et al*. [12] investigated the effects of trypan blue on human trabecular cells for time-dependant toxicity. Their study was able to show that damage to living tissues occurred after an exposure time of 60 s. The desire to move away from trypan blue, lactophenol blue and other dyes comes from their potential carcinogenic properties and long-term hazards to human health. It is acknowledged that the fixative formaldehyde is also carcinogenic, but this is removed during the staining process when the samples are washed prior to autoclaving. The focus of this study is the stain itself, which remains in the plant tissue after the process has been completed.

The use of an ink–vinegar stain has been proposed as a safer alternative. The chemical composition of the ink–vinegar stain is not stated by [11, 13], although a key component is vinegar or acetic acid (CH_3_COOH). The constituents of commercially available fountain pen ink were reported by [14] as being primarily elemental carbon (48%), high carbon-containing organic compounds (23 %), sodium sulphate (16 %), calcium sulphate (7 %), potassium sulphate (4 %) and 1 % ‘other’ (iron sulphate, copper and zinc). This approach to the staining of fungal components was reported previously, two decades ago, by Vierheilig *et al*. [11]. They initially compared the staining of root samples from several species of arable crop, including beans (*Phaseolus vulgaris*), barley (*Hordeum vulgare*), cucumber (*Cucumis sativus*) and wheat (*Triticum aestivum*). Vierheilig *et al*. [11] reported that the stain was of sufficient quality to enable he identification of a difference in root fungal components between the crops studied. The use of an ink–vinegar stain has not, however, gained in popularity in the years that followed the publication of Vierheilig *et al*.’s work [11]. The reasons for this are not entirely clear. Extracellular hyphae from *Rhizoctonia cerealis* inoculation were stated as being observable on wheat roots, but the authors do not present the data in any further detail. Another potential factor that may explain the lack of more wide-scale adoption is that in subsequent years, focus shifted toward the immunological identification of characteristic intracellular root cortical fungal structures [13].

Immunohistochemical (IHC) methodologies are advantageous due to their higher specificity and the reduced damage caused to histological architecture [15]. When processing larger volumes of samples, however, chemical staining is preferred due to higher throughput, ease of use and fewer training requirements for the user. Both approaches confer advantages and disadvantages. Meanwhile, the ink–vinegar stain method originally reported by Vierheilig *et al*. [11] has remained largely ignored. The re-evaluation and further development of this method are therefore overdue.

The present study compares the efficacy of trypan blue and Sheaffer blue ink as stains of intracellular AM fungal components in the root sections of two varieties of winter wheat (Zulu and Siskin). The focus is on image clarity, quantifiable structures (arbuscules and vesicles) and the potential for the substitution of a hazardous staining material with a safer alternative.

## Methods

### Seed variety

Winter wheat (variety: Siskin), 98 % germination rate, with no chemical pretreatment was supplied by KWS UK Ltd. A second winter wheat variety (Zulu) was sourced from a farm in central Hertfordshire as farm-saved seed. The percentage of organic matter of the adjusted soil was confirmed via modified loss on ignition (LOI) methodologies obtained from Myrbo *et al*. [16], using 5 g of adjusted soils heated at 400 °C for 12 h. Soils were adjusted through the addition of J Arthur Bowers multipurpose compost to correspond with he measured percentage of organic matter of field-tested farm topsoil equating to 5 %.

### Growth conditions

Individual seeds were introduced into 300 g of adjusted, pre-purchased, top soils (J Arthur Bowers) and kept in controlled growth room conditions at 25 °C, 1770 lm and a humidity of 35 %.

### Sample preparation and staining

Intracellular root arbuscules and vesicles were examined after the roots were left fully submerged in a formaldehyde, acetic acid, alcohol (FAA) and deionized water solution, 10 : 5 : 50 : 35, respectively, for 24 h. Roots were removed and rinsed with deionized water prior to autoclaving. Root systems, containing small quantities of soils, were subject to sonication at 42 kHz for 10 min and rinsed in deionized water. If small amounts of soils still adhered to root systems, a soft fine paint brush was used to remove debris. Root systems were submerged in 5 % hydrochloric acid for 30 min and incubated at 60 °C in a water bath. After cooling to room temperature, root material was sectioned into 1 cm pieces, with adjacent sections subjected to different stains. Five 1 cm root sections were each allowed to stain in 0.4 % trypan blue in phosphate-buffered saline (PBS) (Fisher Scientific) and 10 % Sheaffer blue ink in 25 % glacial acetic acid [11] for 3 min. Samples were produced over a 6-week period from Zulu (*n*=60) and Siskin (*n*=60) wheat varieties in controlled growth conditions. The samples were viewed initially at a total magnification of 40× using a Vickers compound microscope. The counting of stained root vesicles and arbuscules was performed at a total magnification of 100×, and fungal components were counted and recorded with the focus on arbuscule and vesicle quantity. Images of samples were taken with a Bresser HD microscope camera.

### Statistics

Standard errors and means were calculated from raw data for each week of sample collection. Paired *t*-tests were employed for null hypothesis testing of differences between trypan blue and Sheaffer blue staining. Statistical significance was determined by *P* values ≤0.05.

## Results

**Fig. 1.**
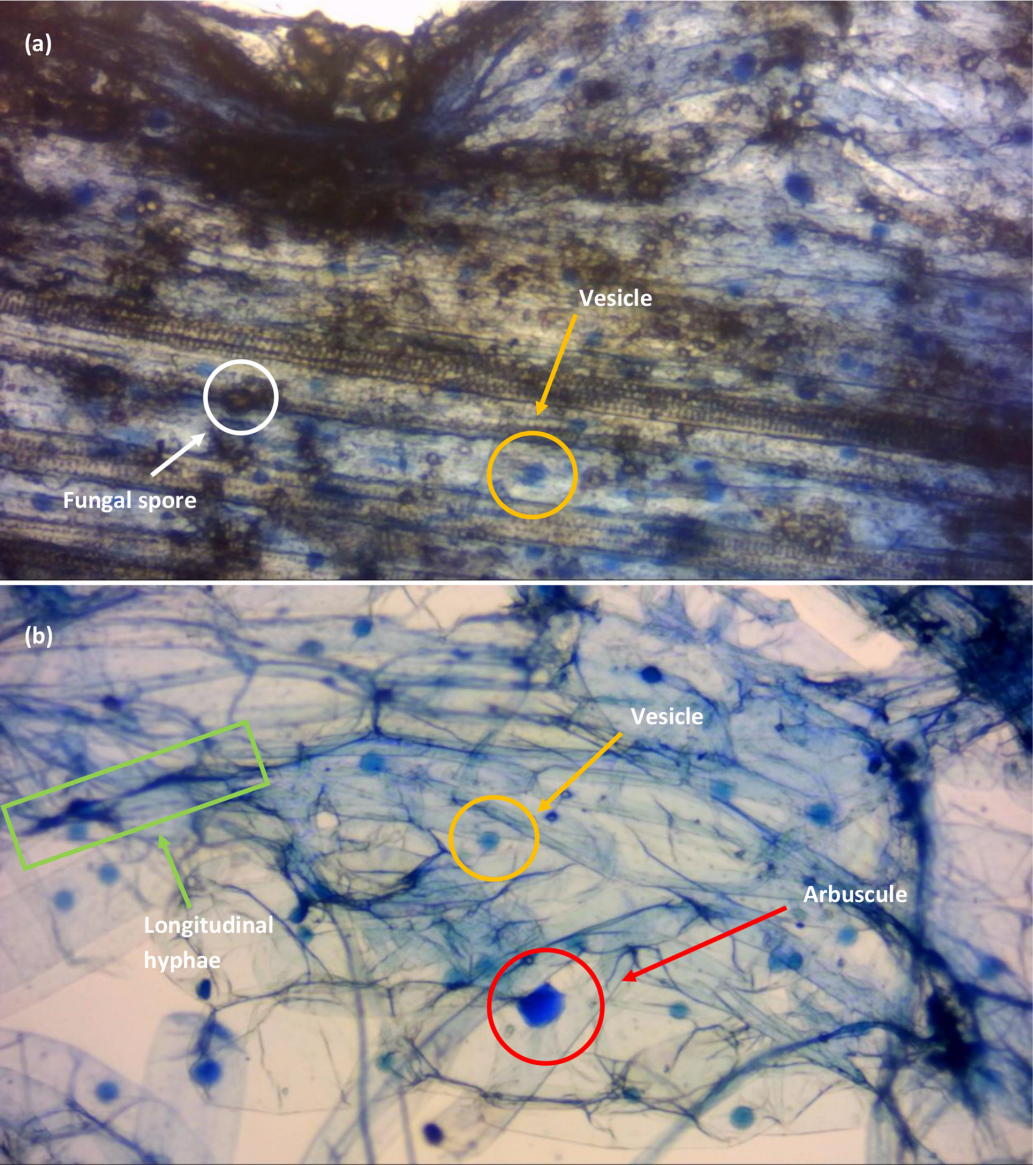
Week-old Zulu variety wheat stained with (a) 0.4% trypan blue in PBS and (b) 10% Sheaffer blue in 25% acetic acid. Fungal vesicles are clearly stained as defined blue spheres, and fungal spores have not been stained and show as transparent brown circles. In (a) trypan blue in PBS was unable to stain longitudinal and radiating hyphal structures. The larger stained structure of (b) was later identified as an intracellular arbuscule after marginal destaining. Images were recorded using a Bresser HD microscope camera under a total magnification of 100× of a Vickers compound microscope.

The difference in clarity of the stained root sample between the trypan blue and Sheaffer blue ink approaches can be seen in Fig. 1a, b. Root-associated fungal spores are shown as light brown spheres in Fig. 1a. These obscure the image for the accurate counting of vesicles and arbuscules, and risk being included within the final count of stained fungal structures. Fig. 1a, b shows the clarity of sample from 1 cm sections adjacent to each other of the same root of the same plant. The degree to which the clearing of roots was carried out was identical for all samples and can be eliminated as a variable – i.e. this was not the cause of the absence of spores in Fig. 1b.

From the samples examined (*n*=120), staining with trypan blue did not produce sufficient clarity (i.e. quantifiably observable fungal root components) in comparison to staining with Sheaffer blue for the two varieties of winter wheat investigated. Quantification of fungal spores is possible from the images presented in Fig. 1a. The observational characteristics are summarized in Table 1.

**Table 1.** A comparative of trypan blue and Sheaffer blue stains for AM fungal root structures and components from stained samples (*n*=120), observable under a Vickers compound microscope at a total magnification of 100×

Observable components	Trypan blue	Sheaffer blue
**Arbuscules**	+/-	+
**Vesicles**	+	+
**Longitudinal hyphae**	−	+
**Radiating hyphae**	−	+
**Fungal spores**	+	−

**Fig. 2.**
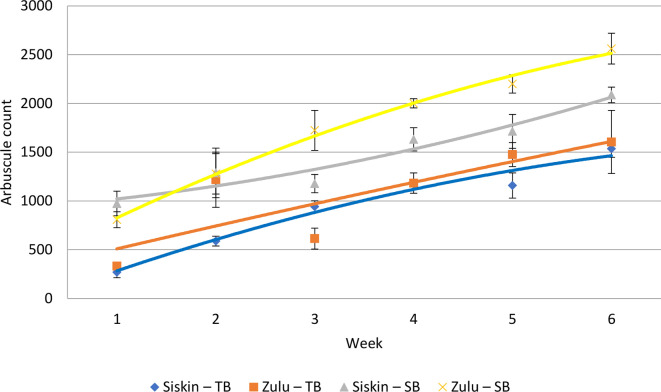
Mean AM fungal root components (*n*=5) per week for comparison between AM fungal stains of Zulu and Siskin varieties of winter wheat using Sheaffer blue (SB) and trypan blue (TB) over a period of 6 weeks (*n*=120). Paired *t*-tests showed significant differences between the staining techniques for Zulu (*﻿P*﻿ 0.003) and Siskin (*﻿P* = 0.0003) varieties. The error bars were constructed from the sem.

Under testing, the null hypothesis, that there are no measurably significant differences between the staining techniques, was not upheld from a paired *t*-test for Zulu (degrees of freedom (df)=5, *t* value=−4.5, *P*=0.003) and Siskin (df=5, *t* value=−7.5, *P*=0.0003) (Fig. 2) varieties of winter wheat. Fig. 2 shows an increase in the difference between fungal components (stained arbuscules) as the age of he root systems increases. The variation in the standard error of the mean (sem) for those samples stained with trypan blue (Fig. 2) reflects the greater variation in the number of AM fungal root structures identified during each sampling week.

## Discussion

The present study has identified a statistically significant difference between staining techniques with respect to observable intracellular root cortical fungal components. The employment of Sheaffer blue ink over trypan blue is more favourable in terms of both improved image clarity and reduction of the user’s exposure risk to chemicals with potential long-term health hazards. As an azo stain, trypan blue is a known carcinogen [17, 18]. Sheaffer blue, on the other hand, is a commercially available pen ink that is much safer. This potentially makes it a far more widely accessible and cost-effective technique.

Although subject to scrutiny in previous studies, the use of ink–vinegar as a staining method has not been adopted widely. In 1998, Vierheilig *et al*. [11] investigated a range of coloured inks for the staining of AM fungus-associated root structures. They commented that ink–vinegar staining allowed the observation of extracellular hyphae on wheat roots inoculated with *Rhizoctonia cerealis*, but did not present the data or images to substantiate the comment in any further detail. Vierheilig *et al*. [11] also used different ink colours, for example black, which were possibly not as effective as the blue ink used in the analysis here. Coupled with a simultaneous shift in interest toward the use of IHC techniques, this may explain the lack of more widescale adoption of the ink–vinegar approach post-1998. Importantly, it contradicts the findings of the current study. Hyphal components were clearly visible in all 60 root samples stained with Sheaffer blue ink. Trypan blue stain, on the other hand, did not provide adequate clarity to allow identification of the same hyphal structures. The clarity of arbuscules and vesicles was hindered using trypan blue due to fungal spores obscuring these structures in the sample. This did not occur when using Sheaffer blue as a stain.

Cottet *et al*. [10] compared 14 different methods of AM fungal staining developed between 1970 and 2014. Each method used a form of fixative solution to stabilize the plant cells, with FAA solution being the most common, while the concentration of trypan blue varied. The microscopy images presented for each method evaluated do not demonstrate image clarity comparable to that in images featuring samples stained with Sheaffer blue (illustrated in Fig. 1b of this study). It is acknowledged that Cottet *et al*. [10] studied the staining of AM fungal arbuscules and vesicles in bryophytes as opposed to the staining of wheat, a monocotyledon angiosperm. The findings of Cottet *et al*. [10], and those of Vierheilig *et al*. [11, 13], who analysed other crop species (namely wheat, barley, beans and cucumber), demonstrate that individual staining methodologies can be applied to a range of plant species. The evaluation of Sheaffer blue as a stain for roots in a broader range of crop types will be investigated in the future.

The clearing of soil materials from roots is an important first step in the preparation of stained samples due to the desired components being potentially obscured by debris. The present study used adjacent root sections. This negates any differences from root clearing. In most cases, roots are cleared with the use of 10 % w/v potassium hydroxide [19]. Whilst this method does remove debris and leave root ready to be processed further for staining, the use of potassium hydroxide reduces the structural integrity of the root cells by chemical degradation of the cell wall [19]. As suggested by Dodd *et al*. [20], the employment of 10 % w/v potassium hydroxide solution should be reserved for root cells that are highly pigmented. The present study utilized plants grown under controlled conditions and did not produce highly pigmented root structures. The data presented by Vierheilig *et al*. [11, 13], Kobae [19] and Cottet *et al*. [10] used plant materials from environmental sources and saw pigmented root cells. In the case of environmental samples, a potassium hydroxide solution would be suitable. More relevant to the present study, root clearing via sonification was sufficient to achieve a level of debris removal to allow further sample processing and staining quantification. By using sonification, the practitioner is removed from a highly corrosive solution, leading to a lower-hazard procedure.

An area of limitation within the method used arises from the manual counting of stained fungal components and the time input required. Although potentially faster approaches exist for the quantification of fungal structures, employing image analysis software, the programs are only able to scan the field of view for objects that are different to the background image. There is a high risk of misidentification and classification of structures and hence this was not considered to be a sufficiently reliable approach for the purpose of the current study.

Ford and Becker [21] produced data, from a study with Wistar rats, indicting that trypan blue had mutagenic and carcinogenic implications for reticuloendothelial neoplasm (RES), predominantly in liver cells. In later years, Kwok *et al*. [22] investigated he toxicity of trypan blue against retinal pigment epithelium (RPE) in cell culture using three concentrations of trypan blue, and discovered reduced cell viability in those treated with trypan blue. The present study suggests a move away from the use of trypan blue staining in plant root cells. No current literature is able to indicate the toxicity or carcinogenic properties of pen ink, indicating a less hazardous alternative to the widely used trypan blue.

In conclusion, the employment of Sheaffer blue staining allows for the effective, safe and low-cost quantification of fungal components in commercially important plant species such as wheat. The manual handling of slides, whether old or new, comes with reduced long-term health risks when stained with Sheaffer blue as opposed to the carcinogenic azo dye trypan blue. Further, the number of fungal components that obstruct viewing are limited, resulting in a more reliable quantification of the established AM fungal infection and symbiosis within the roots of wheat plants.
